# Delayed Disclosure of HIV Status and Lack of Resources Affect Older Persons during Care of Adult Family Members with AIDS-Related Illness in Rural Mpumalanga, South Africa

**DOI:** 10.1155/2020/3430847

**Published:** 2020-06-24

**Authors:** Sphiwe Madiba, Makhosazane Ntuli

**Affiliations:** Department of Public Health, Sefako Makgatho Health Sciences University, Pretoria 0001, South Africa

## Abstract

**Purpose:**

This paper examines the older persons' knowledge of HIV and AIDS and explores the effect of delayed disclosure of HIV status and lack of resources during care.

**Methods:**

The study site was health facilities in Thembisile Hani subdistrict, Mpumalanga Province, South Africa. Older persons aged 60 years and above were selected using purposive sampling for in-depth interviews. Thematic analysis was used to analyse the data.

**Results:**

Providing physical care to sick adults is labour intensive for the already weak older persons. They undertake the caring role within constraints arising from lack of resource such as gloves, diapers, and soap with which to perform the caring role. Taking care of the sick needed resources for specialized care and money for transport to the health facilities. This put a strain on the finances and rendered the older persons food insecure. Furthermore, disclosure of HIV status was delayed, and some older persons cared for the sick adult children without knowing that they were HIV-infected and had AIDS-related illnesses. The nondisclosure of their HIV status by the sick prevented them from taking precautionary measures to prevent the risk of infection during the provision of care. This was heightened by the limited knowledge of HIV/AIDS of the older persons.

**Conclusion:**

Older persons undertake the caring role with diligence under trying conditions due to lack of resources and the nondisclosure of HIV status of the adult children they take care of. Healthcare workers should educate older persons to take preventive precautionary measures when caring for family members even when there is no suspicion of HIV infection. In addition, access to the incapacity grants provided for individuals sick with AIDS-related illnesses could relieve the older persons from financial constraints.

## 1. Background

South Africa has a greater proportion of older persons than any other sub-Saharan country [1]. According to Statistics in South Africa, the older person's population aged 60 years and above rose from 7.1% to 8% between 1996 and 2011 (from 2.8 million to 4.1 million people). In South Africa, the increase in the older person's population is attributed to the shrinking general adult cohort, due to the increased mortality rate amongst children and prime-age adults as a consequence of AIDS as well as a decline in the fertility rate [1, 2]. In most households where older persons have experienced the death of an adult child through AIDS-related illnesses, the deceased adult children were breadwinners. Such deaths change the family structures, which are now without the economically viable middle generation [3–6].

HIV and AIDS affect older persons in a number of ways. As stated, most of the households with an HIV/AIDS person have elderly people who eventually take up the role of caring for the sick adult child. Therefore, older persons have been in the forefront in the care of sick people since the advent of the HIV and AIDS epidemic [7–9]. Moreover, most of such households are female-headed; therefore, the majority of the women become caregivers who undertake the task [1, 2, 5, 10–12].

The older persons continuously care and support individuals infected with HIV amidst different challenges such as limited HIV related knowledge and skills, financial resources, social, and emotional support [8, 13, 14]. There are no specific HIV education and health promotion programmes, which address HIV prevention, transmission, and the protection of older persons from the risk of infection when caring for their sick and terminally ill children. This is as a consequence of commonly held notion where elderly people are minimally at risk of HIV transmission through providing care [8]. Most of the HIV awareness campaigns and programmes do not target older persons, who are excluded from the HIV/AIDS programmes [15]. Similarly, at global level older persons are not targeted as a specific group either in the Millennium Developmental Goals or lately in the Sustainable Developmental Goals [1]. This is despite projections that the population of elderly people over the age of 60 years in Sub-Saharan Africa will nearly double in 2050 [1, 16, 17].

The lack of the involvement of older persons in HIV programmes is now the focus of international policy makers who urge the development of strategies to enhance and strengthen the provision of services to improve quality of life of this population group [18]. HIV education campaigns should not only target persons thought to be at high risk of infection but also facilitate correct knowledge and understanding of the nature and causes of HIV and AIDS for older persons. This could be achieved through the incorporation of older persons as part of the target group in information education communication campaigns [8].

The purpose of the main study, from which this paper emanates, was to develop an HIV and AIDS educational programme for older persons to meet their needs as they care for family members with AIDS-related illness. The study responds to the need to develop HIV and AIDS educational programmes for older persons to include them in the programmes that address HIV and AIDS [19, 20]. Determining what older persons need to know about HIV and AIDS might be useful in utilizing them effectively in HIV prevention, treatment, and support [19]. The focus of this paper is therefore to examine the older persons' knowledge of HIV and AIDS and describe how delayed disclosure of HIV status and the lack of resources affect them during care for those sick with AIDS-related illnesses.

## 2. Methods

### 2.1. Study Design and Setting

The data for this paper were extracted from the second author's doctoral study in public health using a mixed method design to develop an HIV and AIDS educational programme for older persons. The study design, setting, and population is defined in detail elsewhere [21]. This paper focuses on the qualitative data generated in the study to explore how the older persons care for their adult children with AIDS-related illnesses and the challenges they met during care. Integrating elements from both the quantitative and the qualitative data informed the development of a relevant HIV educational programme.

The study site was primary health facilities in Thembisile Hani subdistrict in Mpumalanga Province, South Africa. The subdistrict has 29 health facilities comprising seven 24-hour community health centres, 13 eight-hour clinics, and a level-1 hospital. In addition, the subdistrict is serviced by 9 ward-based outreach teams. The facilities were selected because of the high numbers of older persons that attend the clinics and collect chronic medication.

### 2.2. Study Population and Sampling

The population consisted of older persons aged 60 years and above who collect chronic medication from health facilities. Almost all collected treatment for hypertension, were in a sane state of mental health, and were not critically ill to answer questions. The older persons were selected using purposive sampling. In purposive sampling, only participants who would inform the objectives of the study are selected. In this study, older persons were selected if they answered yes to indicate that they had taken care of an adult child with an AIDS-related illness. They were recruited from the twelve health facilities that formed part of the study setting. A total of 31 older persons were sampled and the recruitment was guided by data saturation.

### 2.3. Data Collection

Data were collected between June and August 2016 by the second author assisted by experienced and trained research assistants. Face-to-face in-depth interviews took place from the selected facilities with older persons using semistructured interview schedule. Although the research assistants were experienced in interviewing, the first author trained them on the objectives of the study and handling sensitive information such as caring for family members with AIDS-related illness in a one-day training session. The interview schedule had open-ended questions and the older persons were asked about (1) the activities they performed during care, (2) the circumstances leading to them having to care for the ill adult child, (3) their thoughts about disclosure of HIV status to parents, (4) the support they received or did not receive from family and others, (5) the difficulties they met during the care they rendered, and (6) what they knew about protecting self from contracting HIV during care. The interviews were conducted in the local language in a separate room to ensure privacy, were audio recorded, and lasted for about 60 minutes.

### 2.4. Data Analysis

The authors adopted thematic analysis to analyse the data. The research assistants who conducted the interviews transcribed the audio recordings of the interviews verbatim in IsiZulu. Each transcript was then translated into English, and the English transcripts were carefully reviewed by the lead investigator who is fluent in the two languages. Line-by-line coding was adopted as the first step of thematic analysis [22]. The authors began by reading a few transcripts independently to identify initial codes that emerged from the data. These codes were used to develop a codebook and the emerging themes were discussed until the authors reached consensus and finalized the codebook. NVivo 10, a qualitative data analysis software was used to code transcripts. Codes were then grouped into categories and emerging themes across the transcripts were identified. Final themes were decided by agreement between the authors.

Trustworthiness was established through credibility, transferability, dependability, and conformability [23]. We used a number of strategies and methodologies to enhance the credibility of the study findings. In addition, we interviewed the participants in the local language, transcribed verbatim to reflect the views of the participants, and spend time in the field to familiarize with the study context and data by taking extensive field and interview notes. Investigator triangulation was employed during data analysis [24], where both authors were involved in the analysis and interpretation of data to reduce investigator bias.

## 3. Results

### 3.1. Description of Study Sample

Thirty-one older persons aged between 62 and 82 years were interviewed. Almost all (30) older persons were female and only one was male. Almost half (15 out of 31) had no formal schooling. All the older persons were from multigenerational households, five of them were living with 9 to 12 household members, 19 had eight children, and only one was living alone at the time of data collection having lost all her children to AIDS-related illnesses. All the older persons had cared for at least one adult child with AIDS-related illnesses, four cared for two sick adults, and one older person cared for three sick adults. The mean age of the sick adults was 33.3 years, range 20–52 years.

The duration of care for most (15 out of 31) sick adults was less than a year, with a range of 6 months to over 24 months. Twenty-four of the sick adults cared for died, and older persons whose children survived reported that they were on antiretroviral treatment and were living positively at the time of data collection. Seventeen older persons did not know about the risk of infection and did not use gloves during the provision of care (Table 1).

### 3.2. Themes

This paper presents the findings from five themes: (1) resource-constrained environment, (2) lack of HIV knowledge, (3) delayed disclosure of HIV status of the child, (4) unconditional acceptance of HIV status, and (5) lack of protective materials.

### 3.3. Resource-Constrained Environment

The older persons performed their caring role under resource-constrained circumstances. They had limited resources and support available to them during the care of their adult children. Taking care of sick adult children need specialized care, money for transport to the health facilities, nutritious food, and protective materials. The older persons found it difficult to provide nutritious food to their sick children and could not afford the frequent need for transportation of the sick to the health facilities.

“*Sometimes I would not have money and he would want fruits only to find that I did not have money to buy them. That was a problem*” (*unknown age*).

An HIV infected person should get food to get better…, but you find that you do not have the money to buy food because the social grant for HIV infected people takes a long time to process. The lack of food hurts the body and the sick person gets weaker (65-year-old).

### 3.4. Lack of HIV Knowledge

The older persons had various understandings of HIV/AIDS; they derived their understanding from what was said in the community and from discussions in the media, such as radio talks. Some had correct information, others had distorted information, whereas others were very confused. Their narratives revealed high levels of ignorance about issues pertaining to HIV and AIDS, including the modes of transmission.

“*The problem is that I do not know how you get infected by HIV, so a person will want to make a tea for me and I am hesitating what if the person passes it to me. What I know is that if I am taking care of an HIV person without having gloves and finding that I have an open wound it can pass to me if the person is also having a wound*.

“*Oh, no…*, *I did not know anything about HIV*; *it was for the first time that I saw someone with HIV*. *I only see them now at the clinic… I learnt about HIV from my sick child*” (*73-year-old*).

“*Sometimes the person got it from the toilet. You can also get it from the toilet!*” (*73-year-old*).

“*Ah... I often hear them say it is bad blood*” (*73-year-old*).

However, some were relatively knowledgeable. It appeared that education and counselling by the health workers to both the sick and the older persons was effective and made it easier for them to understand the role of caring for adults with AIDS defining illness.

“*I say…, there are many ways to get infected with HIV*. *If I can touch infected blood and it happens that I have a cut on my hands I can be also infected*” (*60-year-old*).

### 3.5. Delayed Disclosure of HIV Status

The data revealed that there was a lot of secrecy around HIV and AIDS and the older persons came to know that their adult children were infected with HIV in various ways. Most of the sick adults did not disclose their HIV status to their elderly parents, who got the information from either the clinics, the hospitals, or the doctors when the adult children were already ill.

“*When I got in, the doctor said,* “*should I tell your mother*”*? She* [*the daughter*] *said, she is my mother, she should know my status. I picked it up there and then*” *(65-year-old)*.

“*She was very sick*…, *worse than the first one* [*another child who had AIDS*]; *she asked me to take her to the clinic to fetch treatment. When we came back she said to me* “*at the clinic they said that I am HIV-positive*.” *She asked me not to tell anyone*” (*70-year-old*).

For some of the older persons, disclosure was delayed to the last minute just before the death of their child or sadly from rumours in the neighborhood after the death of their child.

“*He did not disclose, he only disclosed when he could no longer talk properly. He told me on Friday and died on Saturday morning*” (*66-year-old*).

“*Maybe they told my wife and she hid it from me but I never heard anything. But I heard from the people in the neighbourhood that my child was HIV-positive*” (*65-year-old male*).

The data revealed that because some of the older persons had no clue of HIV and AIDS, they did not suspect anything when their children became ill.

“*I did not suspect. When he became seriously ill, I took him to the doctors. I wanted to go with him into the consultation room so that I could hear what was wrong with him. I wondered why he chased me out when I went in with him*” (*76-year-old*).

“*I never suspected because when a person comes home sick you just think about the place that you can take her to, like I took her to the doctor, the doctor referred me to the clinic*” (*age unknown*).

Disclosure is a process and many people disclosed after some time, after they themselves had come to terms with the HIV diagnosis and are ready to disclose. Some sick adults openly disclosed their HIV status to their elderly parents; however, the actual stage at which disclosure took place and the context surrounding disclosure differed.

“*As their parents, I saw them getting sick*, *I saw the other one coughing, getting skinny, and sleeping all the time with no energy. I then asked her, what was wrong, but she did not tell me anything and then she came to the clinic to get tested and got treatment. That is when she came to me and told me that this is between me and her*; *I must not tell anyone. She said to me ‘I went for test and I have found that I am HIV; I got it from my boyfriend*” (*64-year-old*).

### 3.6. Unconditional Acceptance of HIV Status

Disclosure was a painful experience for all the older persons, regardless of the manner in which they were informed. However, those who learned about the HIV status of their adult children became supportive and nonjudgmental, accepted their children, and committed to looking after them.

“*I fainted here at the clinic*…, *I had a heart attack because I never thought my child would have HIV*, *but, after I woke up, the nurse stayed with me and explained everything to me*…, *Yes I was shocked, but I accepted because I got to realize that there are so many other parents who are experiencing this disease*” (*unknown age, older person*).

To be honest, I did not want her to go to the hospital because I looked after her with my own hands. Even when they came to take her, I was refusing” (65-year-old).

“*I bathed him, dressed him, and did everything for him… I asked him to stop being afraid of me because he can see that he is unable. I am his mother*” (*75-year-old*).

### 3.7. Keeping the HIV Status Secret

The sick adult children did not realize that they were hurting their elderly parents by not disclosing to them. Most wished that their children had disclosed to them.

“*For me it is nice to talk in good time. They should say, ‘Ma I have such an illness* [*HIV*]'. *A parent will not trouble the child*… *She will protect the child from the outsiders*” (*65-year-old*).

“*It is important to tell so that you are able to assist them because they have told you what their sickness is. So, if they hide the disease then you will not know how to help*” (*76-year-old*).

“*It is better if the child talks so that he/she can get help. Isn't it. So he/she can go to the clinic or you as the parent could take him/her to the clinic”* (*65-year-old*).

Some wished to keep their children's HIV status secret from others. They believed that the child's HIV status should be kept secret and by doing so, they perpetuate the secrecy around an HIV diagnosis.

“*It is difficult…, HIV is a disease that has to be kept secret. It is difficult to tell a person that your child has HIV*” (*63-year-old*).

“*It is important to keep it as a secret because my child said I must keep it a secret. If my child can hear from people that I have told, they would not like it*” (*age unknown*).

“*Keep it a secret. Only tell the doctor because the doctor will not go about telling people. You tell a person in confidence, the person then goes about telling other people*” (*68-year-old*).

However, when it came to the issue of disclosure of their child's status to others, there was a difference in opinion. Some older persons did not keep their children's status secret. They reported that they told other people, so that they could benefit from their experiences.

“*What I know is that people do not talk about HIV and AIDS, they are scared that people will say that their children were naughty… I told everybody that my son was HIV-positive… I did not hide it… until he passed on. One day when I was in Church, I stood up and told the people about my child*'*s status and asked them for prayers*” (*69-year-old*).

### 3.8. Lack of Protective Materials

Their illnesses incapacitated the sick adults to the extent that they suffered bowel and bladder incontinence. The lack of protective materials such as gloves, adult diapers, and linen savers was a challenge for the older persons. The lack of gloves to protect themselves from the possibility of becoming infected from such excreta and wound exudates exposed them to the risk of being infected. In addition, the constant washing of the linen soiled by the bowel and urine incontinence was often performed without soap and gloves. The risk of infection with HIV was apparent from what some of the more knowledgeable participants said.

“*I did not protect myself because I did not know how HIV could be transmitted to me…*, *No one ever taught me*” (*74-year-old*).

Others did not know how HIV is transmitted and thus took no measures to protect themselves from possible HIV infection.

“*I did not know anything. It is only after she passed away that I was informed that an HIV-positive person should not be handled without gloves. I did not know anything before that*” (*76-year-old*).

“*The nurses told us to always wear the gloves when we were assisting him, but they never really told us the actual reason, they only told us later when he had already died. They told me that I had to protect myself*” (*68-year-old*).

Some found out about the need to use gloves only after they had already taken care of their adult children without gloves.

“*They taught me what to do so that I would not be infected. At first I did not know that I had to protect myself*…, *I was just touching and washing her without gloves. I came to the clinic and they taught us*…, *now I have knowledge*” (*age unknown*).

“*I did not know…, there were no gloves. The only person who knew that I should be wearing gloves, is my younger daughter. She told me that I am not supposed to touch her sister with my bare hands, she said I should have gloves. She bought them after she had told me but I did not take notice*” (*age unknown*).

Those who learned about the use of gloves after they had already taken care of their sick children did not know that they could be exposed to HIV infection if they did not use gloves.

“*We only noticed that at the hospital people were wearing gloves but we did not know why. At home, I did not see the need to use gloves because I did not have any*” (*65-year-old*).

“*I did not know anything. It is only after she died that I was informed that an HIV positive person should not be handled without gloves. I did not know anything before that*” (*76-year-old*).

Those who knew they needed to use gloves for protection after they had taken care of their sick children or after their children died got to know that they were exposed to HIV infection. The fear of infection led them to offer themselves to be tested for any possible illness after the death of their adult children.

*“I fell ill and I thought of the incidence of my neighbour who took care of her child who eventually died. After a short while, my neighbour also died. I thought the same might happen to me. I went to the clinic and requested them to check me everything, everywhere, to check me everywhere*” (*73-year-old*)

“*Saying that I do not sleep around comes last because people say this thing* [*HIV*] *stays in the blood for years without knowing. When my child was sick I checked it once; I wish I checked it three times*” (*70-year-old)*.

## 4. Discussion

Since the advent of HIV epidemic, the caring of infected people has become primarily the responsibility of elderly women. When the adult children got sick from AIDS-related illnesses and the illness advanced, the elderly parents became the primary caregivers. They assumed the responsibility for the care of their sick HIV/AIDS children as it has been a long-standing societal expectation in most African countries that they would do so [20, 25, 26].

In the current study, the caring for sick adults occurred in a context where the older persons had little knowledge concerning HIV/AIDS. Their ignorance perpetuated some of the myths and misconceptions surrounding HIV and AIDS in communities. In most African countries the family caregivers, including the elderly parents, are not trained and lack the skills required for caring for people with AIDS-related illnesses [11]. There has been widespread mass media coverage aimed at the general population but none aimed at older persons. Nevertheless, the older persons did their best to provide for the physical and emotional needs of their sick adult children. This was regardless of their educational level and knowledge of HIV/AIDS and the fact that most of them are over 70 years of age. Providing physical care such as bathing, turning the sick adult, and supporting them to the toilet is labour intensive for the already weak older persons who ended up being physically ill not withstanding their existing medical conditions. Eighteen older persons reported that caring affected their health.

The older persons performed their caring role under resource-constrained circumstances living in multigenerational households. In the South African context, multigenerational households are constituted by elderly parents, married or unmarried adult children, and grandchildren staying in the same household [7]. This living arrangement is a result of the general poverty of the community and the lack of employment opportunities for the adult children, which leads to their coresiding with the elderly [4, 27]. Multigenerational households result in the older persons taking the responsibility to provide care to their adult children infected with HIV/AIDS including their orphaned grandchildren using their pension money [28–30]. The older persons in rural areas are faced with a high rate of unemployment of their adult children, even before they take ill, and they have to depend on their pensions to provide care. Schatz et al. [5] found that pensions for the elderly play a very important role in female-headed households affected by HIV and AIDS. However, in countries where there are no social pensions for older persons, the effect of HIV/AIDS is so intense that some have no alternative but to become beggars in order to make ends met [31, 32].

In this study, older persons had limited resources to care for the sick as well as the entire family. The older persons and their households were food insecure; more than half of them reported going to bed hungry even though they spent almost all their pension money on food. The frequent need for transport to accompany the sick to health facilities for follow-up and treatment refill put a strain on the finances of the older persons. Taking care of the sick needed specialized care and money for transport to the health facilities and resources to perform their caring role. The lack of resources such as gloves, diapers, and soap with which to perform the caring role was a challenge. This led to the risk of the possible transmission of the HIV infection.

A lack of resources was evident in the current study, as in others. About a quarter of the older persons were faced with the challenge of providing care to those with AIDS-related illnesses without the necessary resources [32]. The findings are consistent with those reported in a study conducted in similar rural settings in Vhembe District in South Africa. The elderly carers lacked resources during the care for people with AIDS-related illnesses [33]. Consistent with other studies, the elderly carers did their best under difficult and trying circumstances to provide physical care and ensure the hygiene of their children [20, 34].

As stated, some of the older persons in this study had limited knowledge of HIV/AIDS and were therefore unable to take precautionary measures to prevent their being infected during the provision of care. It should be noted that older persons are at risk of becoming infected because preventive information on HIV/AIDS does not reach them [13, 31] as most preventive messages target the youth, excluding the elderly. Similar findings were reported by Sefasi [32] that three-quarters of the older persons who took care of people with HIV/AIDS had limited or no information on the disease. This lack of knowledge increased their risk of HIV infection, especially when there was also lack of resources such as gloves, diapers, and linen savers. Caring activities such as bathing and washing soiled linen were often performed without wearing gloves, thus exposing the older persons to the risk of HIV infection.

Furthermore, some of the older persons cared for their sick children without knowing that they were HIV infected and had AIDS-related illnesses as their children did not disclose their HIV status at the beginning of their illness. For some of the older persons, disclosure did not occur in a formal way; instead, they learnt about the HIV status of their children when the diagnosis was made at the health facilities at an advanced stage of the illness. Literature has shown that people living with HIV take a long time to disclose to their families, especially to their mothers, because they want to protect them from the emotional burden. Others fear for their mother's health status [35–37]. Furthermore, the context in which disclosure should occur influences disclosure to other people; for many people living with HIV, disclosure occurs when they feel that it is safe to do so [36].

Nevertheless, the older persons in this study were of the view that feelings of guilt, shame, and self-blame led to the nondisclosure early in the illness of their children. This is suggestive of self-stigmatization, which is characteristic of being diagnosed with HIV/AIDS, as highlighted in other studies [38, 39]. They believed that their children felt too embarrassed to disclose, considering that HIV is an illness that is spread through sex, and they did not want their parents, families and the community to judge them.

The nondisclosure of their HIV status by the sick adult children exposed the older persons to the risk of HIV infection, considering that disclosure did not occur at the beginning of the illness [40]. Normally one would not wear gloves or take preventive precautionary measures when there is no suspicion of HIV infection even when the resources are available when caring for family members. Studies have reported that the strength of the sense of parental obligation outweighs the fear of infection, and the elderly parents carry out their caring role regardless of the consequences of providing personal care to those who are sick with AIDS-related illnesses [11, 41]. In the current study, even when the older persons knew that their adult children had AIDS-related illnesses and knew that they had to use gloves, they often did not use them when they believed that the use of gloves would be interpreted as repulsion by the sick.

Ignorance about the risk of being infected during the caring activities that the older persons performed also exposed them to the risk of HIV infection. Some of those who knew about the HIV status of their children and the need to use gloves found it very difficult to actually use the gloves. The decision not to use gloves was informed by a lack of understanding of the mode of transmission of HIV. These findings underscore the importance of educating the older persons who may do caring for people infected with HIV/AIDS. A study conducted in Vhembe District in Limpopo also showed that older persons are not familiar with ways of protecting themselves from the risk of HIV infection [33].

## 5. Limitations

The study has some limitations; in line with qualitative inquiry, the study findings cannot be generalised to older persons caring for sick adult children in other study settings. The study involved older persons in rural setting; therefore, the findings are limited to populations of older persons in similar settings. In addition, the fact that older persons with chronic illnesses were sampled and those without were not included and may have other insights further limits generalization. The study assumes that the older persons' knowledge and understanding of HIV/AIDS will improve their attitude and ability to care for family members with AIDS-related illness after being trained. The limitation is that the educational programme may not be adopted and implemented by the healthcare providers in health facilities as recommended by the study. Furthermore, older persons in urban settings might fare better concerning material resources and HIV knowledge. Therefore, they might experience caring for adult children differently.

## 6. Conclusions

As much as the older persons undertake the caring role with diligence, it is never without challenges, some of which arise from their lack of resources with which to undertake the caring role. The older persons and their households were food insecure because taking care of the sick needed money for transport to the health facilities which put a strain on their finances. Of note is that providing physical care to sick adults is labour intensive for the already weak older persons whose health was negatively affected. Health facility staff should advise older persons to access the incapacity grants provided for individuals sick with AIDS-related illnesses by the Department of Social and Security Services. The grant money could be used for resources, food, and transport for follow-up clinic visits.

The older persons worked under trying conditions and handled an unknown illness, due to a number of factors, including the nondisclosure of HIV status of the adult children they take care of. Consequently, some cared for their sick adult children with no information on the disease. Healthcare professionals have a critical role to play in educating older persons to take preventive precautionary measures when caring for family members even when there is no suspicion of HIV infection.

## Figures and Tables

**Table 1:** Sociodemographic, health, and caring characteristics of older persons.

Variables	Categories	Frequency	Percentage
Age group	62–69	19	61.3
	70–82	12	38.7
Education attainment	No formal schooling	15	48.4
	Primary education	13	41.9
	Secondary education	3	9.7
Age of sick adults cared for	20–30 years	10	32.3
	31–35 years	5	16.1
	36–52 years	8	25.8
	Do not know	8	25.8
Sex of adult child with AIDS-related illness	Female	21	70
	Male	9	30
Number of sick adults cared for	1	26	83.87
	2	4	12.9
	3	1	3.23
Duration of care for sick adults	Less than a year	15	48.4
	1-2 years	6	19.4
	>2 years	7	25.8
	Do not remember	2	6.4
Outcome of care for sick adults	Died	24	80.0
	Alive	6	20.0
Knew about risk of HIV infection during care	No	17	54.8
	Yes	14	45.2
Knew about the use of gloves during care	No	16	51.6
	Yes	15	48.4
Used gloves during care	No	17	56.7
	Yes	13	43.3
Number people depending on older person	1–5 persons	22	73.3
	>5 persons	8	26.7
Skipped a meal in the past month	No	16	51.6
	Yes	15	48.4
Went to bed hungry in the past month	No	17	54.8
	Yes	14	45.2
Spending pattern of older persons	Food	14	45.2
	Food and medication	3	9.7
	Food and funeral policy	9	29.0
	Food and electricity	5	16.1
Child support grant for grandchildren	No	8	30.8
	Yes	18	69.2
Knowledge on caring for sick adults with HIV/AIDS	I do not have knowledge	14	45.16
	I have adequate knowledge	8	25.81
	I need information	9	29.03
Self-reported health status	Good health	12	38.7
	Poor health	19	61.3
Effects of caring on health	Did not affect health	13	41.9
	Affected health	18	58.1

## Data Availability

The datasets analysed during the current study are available from the corresponding author upon reasonable request.
